# Pathways to suicide attempts in rural Uganda: a qualitative study of survivors, family members, and healthcare providers, and community health workers

**DOI:** 10.1080/00207411.2026.2704372

**Published:** 2026-07-19

**Authors:** Yang Jae Lee, Mildred Asasira, Amir Hassan, Rauben Kazungu, Ryan M. Aud, Elizabeth Moffett, Loukas Kang, Katherine Ager, Mugamba Tribius, Robert Rosenheck, Patrick J. Raue, John C. Fortney, Jurgen Unutzer, Alexander C. Tsai

**Affiliations:** a Department of Psychiatry, University of Washington, Seattle, WA, USA; b Empower Through Health, Jinja, Uganda; c Department of Psychiatry, Massachusetts General Hospital, Boston, MA, USA; d School of Medicine, Virginia Commonwealth University School of Medicine, Richmond, VA, USA; e Williams College, Williamstown, MA, USA; f Department of Psychiatry, Yale University, New Haven, CT, USA; g Center for Global Health and Mongan Institute, Massachusetts General Hospital, Boston, MA, USA; h Harvard Medical School, Cambridge, MA, USA; i Department of Psychiatry, Mbarara University of Science and Technology, Mbarara, Uganda

**Keywords:** Suicide, Africa, qualitative research, Uganda, rural health

## Abstract

**Introduction:**

Suicide is a critical public health issue worldwide. Risk factors are exacerbated by cultural stigma, socioeconomic hardship, and limited mental healthcare access. However, few studies have examined the lived experiences of suicide attempt survivors in Africa to probe the underlying drivers.

**Objectives:**

To explore the lived experiences and perceived drivers of suicide attempts in rural Uganda among individuals with lived experience, their families, healthcare providers, and community health workers.

**Methods:**

We conducted in-depth qualitative interviews in Buyende District, Uganda from June to August 2023 with a purposive sample of 54 participants aged 23 to 70: people who had survived a suicide attempt (*n* = 18) and their family members (*n* = 17), healthcare providers (*n* = 10), and community health workers (*n* = 9). Interviews were recorded, transcribed, translated, and analyzed using a thematic framework method.

**Results:**

Four main themes emerged as critical factors leading to suicide attempts: (1) familial and spousal conflict, involving relationship breakdown, resource denial, and social isolation; (2) financial stress, including chronic poverty, acute debts, and perceived socioeconomic failure; (3) mental illness and substance use, notably depression, psychotic symptoms, and alcohol use complicating existing psychological distress; and (4) religious and spiritual influences on the attribution of suicidal thoughts.

**Conclusion:**

In this qualitative study from rural Uganda, suicide attempts across were perceived to result from intertwined interpersonal, economic, mental health, and spiritual factors. Participants rarely framed suicide as the result of a single cause, but rather as a response to cumulative and unresolved distress. Moments of missed support suggest opportunities for targeted intervention.

## Introduction

Suicide is a major public health concern in low and middle-income countries (LMICs) (Best et al., 2018; GBD 2021 Suicide Collaborators, 2025). Eastern sub-Saharan Africa bears a disproportionate burden, recording the world’s highest suicide rates, particularly among men, reaching 19.9 per 100,000 in 2021, with an overall rate of 12.2 per 100,000 across both sexes, compared with global rates of 9.0 per 100,000 (GBD 2021 Suicide Collaborators, 2025). In Uganda, suicide is a growing public health crisis, exacerbated by widespread poverty, limited access to mental healthcare services, and mental illness stigma (Kigozi et al., 2010; Lee et al., 2024).

Multiple structural, cultural, and societal factors contribute to Uganda’s high population suicide rates. Socioeconomic instability, including poverty, unemployment, and limited access to education, exerts significant psychological strain, particularly in rural communities where mental health services are scarce (Lund et al., 2011; Mushavi et al., 2020; Tsai et al., 2016). Deep-seated cultural and religious taboos further discourage open discussions about mental health, reinforcing stigma and deterring individuals from seeking help (Kitafuna, 2022; Rasmussen et al., 2019). Additionally, social isolation, familial pressures, and stigma create further barriers to suicide prevention (Motillon-Toudic et al., 2022). Globally, suicide attempts and deaths are understood to arise from multilevel influences, including individual factors (e.g., mental disorders and social isolation), and structural determinants (e.g., poverty and constrained access to care), with protective influences such as social connectedness and access to timely, effective support (Na et al., 2025; Turecki & Brent, 2016).

Suicide remains criminalized in Uganda, and mental healthcare providers are largely divided about the appropriateness of this law (Hjelmeland et al., 2012). Beyond legal constraints, religious interpretations - particularly within Christianity, which is the dominant religion in Uganda - frame suicide as a grave sin and as a breach of divine law (Mugisha et al., 2013). Traditional African beliefs also influence public perceptions of suicide, with some communities viewing suicide as a spiritual abomination that disrupts ancestral harmony (Knizek et al., 2021). Among the Acholi of Northern Uganda, for example, suicide is perceived as a severe social transgression that necessitates community ritual cleansing to restore communal harmony (Mugisha et al., 2018). Similar cultural narratives have been observed in Ghana, where suicide is perceived not only as an individual moral failing but also as a social transgression that harms the collective well-being of the community (Osafo et al., 2011). These perspectives reinforce stigma, social exclusion, and barriers to seeking mental health support, highlighting the importance of culturally responsive suicide prevention strategies.

A critical barrier to developing effective, evidence-based interventions to prevent suicide in Africa is the lack of data. Suicide incidence data are available for fewer than one-third of African countries, leaving large knowledge gaps about the epidemiology, correlates, impacts and experiences of suicide across the continent (Mars et al., 2014). In Uganda, there is limited context-specific research on lived experiences of people who survive their attempts to die by suicide, limiting our ability to understand how social, cultural, and psychological factors interact to shape suicide risk. To address these knowledge gaps, we investigated the experiences of adults in Buyende District, Uganda who survived their suicide attempts. Through in-depth interviews (IDIs) we sought to identify key patterns and specific triggers within the local context.

## Methods

### Study site

This qualitative exploratory study was conducted in Buyende District, a rural area of eastern Uganda with a population of approximately 450,000 people (Uganda Bureau of Statistics, 2024). Buyende District was selected for this study because it is representative of many rural areas in Uganda, with most residents engaged in subsistence farming (Uganda Bureau of Statistics, 2022). Previous research in this region has highlighted the complex healthcare-seeking behaviors of local residents, including the use of both traditional and biomedical care options (Lee et al., 2019).

### Study design and data collection

We conducted one-on-one IDIs between 05/06/2023 and 22/08/2023 to explore individual experiences and perspectives in a purposive sample of adults who had survived a suicide attempt, their family members, healthcare workers, and community health workers. The interview guides were created by the research team and pilot tested before the start of data collection. We recruited participants through multiple pathways. People who had survived their suicide attempts were recruited either through an ongoing cohort of area residents who had agreed in previously conducted studies to be contacted for future research or through volunteer community health workers. We also interviewed, separately, their family members (with the exception of one man who did not tell anyone about his attempt). Healthcare workers and volunteer community health workers were chosen via convenience sampling from health center rosters in the area.

We considered for inclusion adults aged 18 years or older who resided in the Buyende District and who had made at least one suicide attempt in their lifetime. We interviewed family members if they were directly related to someone who had attempted suicide and were aware that the suicide attempt had occurred. Healthcare workers were eligible if they had one year of education past secondary school (i.e., certificate) and had experience caring for at least one patient with a prior suicide attempt. Volunteer community health workers were eligible if they were part of the Village Health Team (VHT), which is a cadre of community-based volunteer health workers in Uganda who are selected by their communities and trained to provide basic health education and referrals to formal health facilities, and had experience caring for people who had attempted suicide (Mays et al., 2017). Written informed consent was obtained from all study participants.

Men and women research assistants with backgrounds in social science and/or public health conducted the interviews after completing training in qualitative methods and research ethics. Three Ugandan research assistants who conducted the interviews were Ugandan, and two were master’s students and another was an undergraduate student. They were all fluent in both English and Lusoga (the local language). For some interviews, Five U.S. research assistants (either medical or undergraduate students) observed and assisted with audio recording but did not directly participate in the interviews. Interviewers used qualitative interview guides with open-ended questions and probes designed to elicit detailed narratives about the experiences, circumstances, and emotional contexts leading up to suicide attempts. Interview questions were intentionally open-ended, enabling the team to collect data about specified topics while allowing unanticipated themes to emerge. The interview guide (Supplementary File 1) was designed to explore multiple domains related to suicide attempts, including precipitating factors, lived experience, and post-attempt reintegration and stigma. The present analysis focuses exclusively on questions related to pathways leading to suicide attempts; other domains are analyzed and reported separately.

The interview process began with VHTs visiting participant homes twice: first to confirm availability and willingness to participate, and again on the interview day to verify inclusion criteria and introduce the research team. Interviews were conducted at the participants’ place of residence or work. In cases where both the person who survived the suicide attempt and a family member were available at the same visit, we conducted the interviews in different parts of the property to maintain privacy. Each interview lasted 30–60 minutes. All interviews were audio-recorded using password-encrypted tablets and uploaded to a secure database accessible only by the research team. They were then translated and transcribed simultaneously into written English from Lusoga within 1 week of the interview. Data collection proceeded until content saturation was achieved. We assessed saturation iteratively during data collection through rapid review of transcripts and analytic memos; we operationalized thematic saturation as the point at which successive interviews did not yield new codes or thematic categories, at which point the team confirmed that further interviews were unlikely to meaningfully expand the codebook. Translations and transcripts were produced by bilingual Ugandan research assistants and underwent quality checks (including spot-checking against audio for roughly 10% of transcripts) by a second Ugandan team member to ensure linguistic and cultural accuracy.

### Ethical considerations

All research procedures were approved by the institutional review boards (IRBs) of Yale University in the U.S. (2000034605) and The AIDS Support Organization in Uganda (TASO-2023-222). Consistent with national guidelines, we obtained clearance to conduct the study from the Uganda National Council for Science and Technology (SS1860ES). This study was conducted in accordance with the principles of Declaration of Helsinki.

Prior to each interview, participants were informed of their right to privacy, the voluntary nature of participation, and the specific circumstances under which confidentiality could be breached - such as in the event of acute suicidality. In such cases, the study protocol emphasized a collaborative approach: participants would first be encouraged to voluntarily involve a trusted family member or provider. If a participant declined and the risk was deemed imminent, a non-collaborative breach could be initiated in accordance with safety protocols. All participants provided written informed consent; those who could not read or write signed with a fingerprint in the presence of a cosigning witness.

A mental health safety protocol was also implemented for participant protection. In cases of passive suicidal ideation, participants were offered a referral to a Psychiatric Clinical Officer (PCO) at the end of the interview and provided with a 24/7 toll-free number for Healthhotline, operated by the Communication for Development Foundation Uganda (CDFU). In two instances, participants expressed acute suicidal ideation. In both cases, interviews were paused and the PCO was contacted immediately to assess risk. Both participants voluntarily agreed to be evaluated by the PCO, and no involuntary or non-collaborative breaches of confidentiality were required.

### Data analysis

We used the framework method (Gale et al., 2013) to inductively identify recurring themes. Common themes found in each interview were labeled with specific codes, with each interview’s analysis conducted line by line and reviewed by at least one Ugandan and one U.S. research assistant. Codes were collaboratively defined and compiled into a code book. Specific quotes were identified with multiple interviews supporting each code identified. This framework was continuously modified by finding common themes after each batch of interviews, with data analysis typically occurring every 3–5 interviews. Primary line-by-line coding was completed by 2 research assistants (one Ugandan and one U.S. reviewer per transcript) that were directly involved with the conduct of the interview (Ugandan RA interviewing and American RA assisting with recording), with discrepancies resolved through discussion and, when needed, adjudication by the study investigators. We managed coding and framework matrices using encrypted digital spreadsheets (e.g., Google sheets) and maintained the evolving codebook in a shared document; no dedicated qualitative analysis software was used. When new codes were developed, the team revisited earlier transcripts to apply these codes consistently across the dataset.

To ensure rigor, we used multiple coders with different cultural perspectives and verified interpretations through team discussions. Each transcript was reviewed by two team members who familiarized themselves with the content before applying codes to significant passages. When questions arose about cultural or linguistic interpretation, Ugandan team members ensured accurate understanding of participants’ meanings in their cultural context. Regular team meetings were held to compare codes and achieve consensus on application of these codes for subsequent transcripts. Team discussions to refine codes and interpret findings were conducted through daily meetings and shared editing of the codebook/framework matrices.

We organized our findings by grouping related codes into distinct categories, which formed the basis of our analytical structure. This structure was continuously refined as we analyzed additional transcripts, incorporating new codes as they emerged. The final step involved transforming our analytical framework into a hierarchy of themes and sub-themes. This process required a comprehensive review of our established categories, in which we identified underlying patterns and concepts that linked multiple categories together.

## Results

Altogether, we conducted IDIs with 54 participants: 18 people who had survived a suicide attempt, 17 family members, 10 healthcare workers, and 9 VHT members. Of the 18 suicide attempt survivors interviewed, most were men (*n* = 16), engaged in subsistence agriculture as a primary occupation (*n* = 16) (Table 1). Most were married or cohabitating (*n* = 16), and educational attainment varied from no formal education to O-Level (ordinary level; lower secondary education completion in Uganda). Most (*n* = 10) reported a single suicide attempt, with 3 participants reporting two attempts, 1 participant reporting 3 attempts, and 2 reporting 5 or more, 2 participants declined to answer how many suicide attempts that they had. Poisoning was the most commonly reported method (Table 2). No participants refused to participate in the study. Through inductive analysis, we identified four themes characterizing the circumstances around suicide attempts as perceived by study participants, their family members, and VHT and health care workers with experience caring for patients in the region: familial and spousal conflict, financial stress, mental illness and substance use, and religious and spiritual influences. Although suicidal behavior is criminalized in Uganda, legal consequences were not commonly described as a direct driver of attempts in participants’ narrative of pathways. However, criminalization may still shape disclosure and help-seeking and is addressed in the Discussion.

### Theme 1: familial and spousal conflict

Study participants commonly identified familial and spousal conflict as a contributor to suicide attempts. These conflicts were frequently co-occurring with disputes about finances and other resources. Some participants described the financial dispute as being symptomatic of the relational dispute. For example, one survivor described this intersection when he was a minor:

I used to look on how the man that my mother claims to be my dad, the way he mistreated me, reaching an extent of not even giving me clothes and not paying my school fees…. People kept telling me to move to the clan that I belong to, and when all this continued, I tried even still asking my mum, but she kept on denying. That’s when I had thoughts of getting a rope.—Survivor, Man, 36 years

Other participants described the financial dispute as being a trigger for the relational dispute. One mother described how a monetary conflict with her son’s father precipitated her son’s attempt:

His father said to let him die and to leave the father with the money. So that is where my son’s anger came from, and it was the main reason he wanted to kill himself.—Mother of survivor, 52 years

Community members also described how perceived favoritism in polygamous or blended families could create deep emotional distress and contribute to suicidality. One community health worker offered this interpretation:

Then the other reason is giving birth to children from different women. You take the children of one wife to school but then the children of the other wife are not going to school. This might cause someone to say, Why don’t I just kill myself since he’s not taking my children to school?—Community Health Worker, Male

Several survivors described multiple forms of spousal conflict simultaneously co-occurring. One participant’s account illustrated how sexual rejection, substance use, and emotional abuse combined:

She refused to have sex with me completely, and that caused a lot of pain. She would go and drink alcohol, come back and abuse me, and command me to sleep somewhere else. She came up with the story that I was HIV positive. That’s the reason why she couldn’t have sex with me anymore.—Survivor, Man, 70 years

Our data reveal how familial and spousal conflicts typically involved multiple, interacting forms of distress rather than single triggering event. Family disputes often centered on financial issues and resource denial, while relationship breakdowns frequently involved infidelity, rejection, and subsequent isolation. The accounts demonstrate how these conflicts developed over time, with communication breakdowns and social withdrawal often preceding suicide attempts. Across participant groups, relationship conflict was described as both a chronic stressor and a proximal trigger: survivors more often emphasized spousal conflict (e.g., infidelity, rejection), whereas family members and community health workers frequently highlighted patterns of resource denial, perceived favoritism within polygamous or blended families, and strained parent-child relationships. Participants commonly described conflicts as escalating over time, with a perceived loss of belonging and support preceding attempts.

### Theme 2: financial stress

The psychological distress related to financial precarity was commonly identified as a precursor of suicidal thoughts, both by people who had survived their suicide attempts and by their family members. For many male participants in particular, financial stress was not only a matter of economic hardship but also a threat to their identity as providers. The inability to meet expectations around provision and fatherhood surfaced repeatedly in their narratives, suggesting that gendered social roles may influence how distress is experienced and expressed.

Looking at my children being home while other kids are going to school, and searching for what to eat, can make any man think. Whenever you don’t have money and you have very many children, you think a lot on how to survive. The children need to go to school, eat, and as well get other basic needs which I cannot afford.—Survivor, Man, 31 years

Community members also described how persistent economic hardship and the inability to improve one’s financial situation could lead to psychological distress and suicidality:

Earnings, someone wants to be with money and be in a good situation at home but it’s not possible. He searches and searches (for money) but finally fail. It stresses out people regarding the way we’ve been hearing people say that those that want to commit suicide have such thoughts.—Community Health Worker, Man

Family members provided additional insight into how financial stress created a sense of hopelessness:

Sometimes those thoughts come to an individual because of the bad financial situation they are going through. Sometimes they see that everything of theirs has paused. And there is nowhere to run to or where to get help. For example, help for where to get food and to help his/her family. And that may cause someone to feel sad.—Wife of male survivor, 25 years

The data demonstrate how financial stress operated through multiple pathways to increase suicide risk. The combination of chronic poverty with acute financial crises, such as accumulated debts, was particularly stressful for the participants in our sample. Financial precarity was discussed by survivors, family members, and community health workers, often in connection with other themes such as relationship conflict and untreated distress. Survivors, particularly men, frequently framed financial hardship as a threat to their role as providers, whereas family members emphasized the resulting hopelessness and perceived lack of options for assistance. Community health workers described how chronic poverty and repeated financial setbacks could amplify distress and contribute to suicidal thoughts.

### Theme 3: mental illness and substance use

Many participants attributed their suicide attempts to mental health problems. One survivor explicitly attributed his suicide attempt to his inability to resist voices commanding him to hurt himself. He shared:

I feel like my head is on fire and burning, and the voice continues to tell me go and tie a rope around your neck. Go and hang yourself.—Survivor, Man, 45 years

Nearly all the healthcare workers emphasized that psychological distress were common among individuals presenting after suicide attempts, often in the context of relationship conflict:

He thought killing himself was the last option. With his wife having children with another husband, he struggled to cope. He felt it was very, very bad and he wanted to kill himself.—Healthcare worker, Man, 38 years

In addition to mental health problems, substance use emerged as a complicating factor:

Drinking alcohol disturbs the mind and brings you closer to suicide… Sometimes marijuana confuses their heads and then they think of killing themselves.—Community health worker, Woman.

Participants commonly described suicide attempts as arising from acute psychological distress, sometimes involving severe mental health symptoms and often intensified by relationship conflict. Substance use, particularly alcohol, was frequently viewed as worsening emotional instability and reducing inhibitions, thereby increasing the risk of suicide attempts.

### Theme 4: religious and spiritual influences

Religious and spiritual beliefs profoundly shaped how participants understood and responded to suicidal thoughts. Multiple survivors interpreted their experiences through supernatural frameworks:

I will not lie to you, for us the spirits are here, and the people that call the spirits are there.—Survivor, Man, 40 years

In his narrative, he attributed his periods of depressed mood and suicidality to a supernatural cause, which prompted him to seek mental healthcare from a traditional healer rather than from a provider at a biomedical facility. Some participants articulated complex spiritual explanations for their experiences:

It’s all about supernatural powers because something just blocks your mind and starts thinking about unnecessary things. All our thoughts get tied by supernatural powers because we get words from the people that come on us. And another thing—the thoughts that we keep on getting keep on changing every now and then.—Survivor, Man, 63 years

Family members often shared similar supernatural interpretations:

I think that it was just the Devil that caused him to do this because we did not have any fight at all beforehand.—Wife, 23 years

Across interviews, supernatural interpretations (e.g., spirits, witchcraft, or demonic influence) were described by survivors and family members. These beliefs appeared to shape help-seeking pathways.

## Discussion

In this qualitative study of patients, family members, health care providers, and community health workers, we provide the first in-depth examination of the lived experiences of people who have survived suicide attempts in rural Uganda. Our findings reveal the multiplicity of factors such as familial and spousal conflict, financial stress, mental illness, and religious and spiritual influences. Through interviews with multiple stakeholders, we identified how individual, interpersonal, and structural factors can combine to create pathways to suicide attempts in this context.

Relationship conflict emerged as a central pathway to suicide attempts, often escalating in the setting of accusations of infidelity, abandonment, and chronic criticism. These accounts underscore the potential value of family- and couple-level prevention strategies that strengthen communication, conflict resolution, and social support during interpersonal crises. The prominence of financial stress in our participants’ narratives aligns with existing literature on suicide risk in Uganda (Abaatyo et al., 2023; Kinyanda et al., 2011), where socioeconomic instability and poverty exert significant psychological strain, particularly in rural communities where mental health services are scarce (Sweetland et al., 2019; Tsai et al., 2012). Our findings extend this understanding by revealing how financial stress operates through multiple pathways: the immediate pressure of providing for basic needs, accumulated debts, and social comparison with peers. Financial difficulties often parlayed into relationship conflicts, intertwining to create conditions for suicide attempts.

The role of spiritual beliefs in our participants’ understanding of suicidal thoughts reflects broader cultural narratives in Uganda, where traditional African beliefs and Christianity significantly influence perceptions of suicide. Our participants’ attribution of suicidal thoughts to supernatural causes, including spirits and demonic influences, echoes previous research showing how suicide is often viewed as a spiritual transgression in Ugandan communities (Mugisha et al., 2013; Neuner et al., 2012). However, our findings suggest that these spiritual interpretations may influence help-seeking in complex ways, sometimes leading individuals to seek traditional healing rather than biomedical care. This aligns with previous research conducted in Uganda demonstrating the high use of alternative healing methods for both physical and mental illnesses (Lee et al., 2019, 2024, 2025c).

The prevalence of depressive symptoms noted by healthcare workers, combined with the complex interplay of social and economic stressors identified in our study, suggests important implications for primary care settings. While nine of ten healthcare workers in our sample described depression as a common presenting feature among individuals who later attempted suicide, this finding is based on retrospective, anecdotal impressions and should be interpreted with caution. Nevertheless, it highlights a potential opportunity for earlier identification of individuals at risk. Given the stigma around mental health in Uganda (Lee et al., 2024, 2025b), our findings suggest that financial stress and family conflicts might serve as more acceptable entry points for discussions about mental health, as these were readily discussed by participants. Primary care providers might therefore consider incorporating questions about economic and relationship stressors into their assessment of suicide risk. While investigating post-attempt experiences was beyond the scope of this study, the stigma attached to suicide attempts in Uganda warrants consideration. Previous research has documented how suicide is often viewed as both a moral and social transgression in Ugandan communities (Knizek et al., 2021). In addition, because suicide attempts have historically been criminalized in Uganda, the legal context may further shape disclosure and help-seeking, and may have influenced which survivors were willing to participate—consistent with our related qualitative work in the same setting showing that legal norms contributed to bureaucratic delays and occasional police involvement after attempts (Lee et al., 2026). This stigma may have influenced which survivors were willing to participate in our study, potentially excluding those who faced more severe social consequences. Future research should examine how stigma shapes both help-seeking behaviors before attempts and access to support afterward.

This study has several limitations that warrant acknowledgment. Our sample was recruited through healthcare workers and previous research participants, leaving the representativeness of attitudes in rural Uganda recorded in this study unknown. Individuals who have not engaged with formal health systems were clearly under-represented. The presence of both Ugandan and American researchers during interviews may also have influenced how participants discussed sensitive topics, particularly around spiritual beliefs and traditional healing practices. Our sample, 16 men and 2 women, were heavily skewed toward men. Although the population of people who attempt suicide in Uganda is more heavily weighted toward men than women (Kaggwa et al., 2022), our data are missing potentially unique perspectives from women.

## Conclusion

This study reveals how suicide attempts in rural Uganda can emerge from complex relationship conflicts, financial hardship, mental health challenges, and spiritual beliefs. The prominence of family conflicts in our data points to the potential value of family-based interventions that address both relationship dynamics and resource management. Previous research in Uganda has also demonstrated that combining economic support with family strengthening components can improve family cohesion and communication while enhancing family assets and employment, ultimately supporting mental health (Karimli et al., 2024; Puffer et al., 2020). Lastly, our findings regarding spiritual beliefs suggest that effective suicide prevention may require engagement with both traditional healing systems, Christian religious authorities and biomedical care providers. This aligns with findings from our companion study in the same setting, which documented provider openness and barriers to collaborative care across these domains (Lee et al., 2025a). Future research should examine how suicide prevention interventions can effectively integrate attention to financial stress, family dynamics, and spiritual beliefs while respecting local cultural frameworks.

## Supplementary Material

Supp 1

Supplemental data for this article can be accessed online at https://doi.org/10.1080/00207411.2026.2704372.

## Figures and Tables

**Table 1.** Demographic information of people who survived suicide attempts.

Sex	
F	2
M	16
Age	
20–29	3
30–39	5
40–49	4
50–59	4
60–69	1
70 or greater	1
Marital status	
Married or cohabiting	16
Separated	1
Widowed	1
Religion	
Protestant	8
Catholic	6
Muslim	2
Pentecostal	2
Occupation	
Peasant farmer	16
Barber	1
Unemployed	1
Education	
Never attended	4
Incomplete primary	7
Complete primary	4
O-Level	3

**Table 2.** Descriptive statistics.

Number of suicide attempts	
1	10
2	3
3	1
4	0
5 or more	2
Decline to answer	2
Method of suicide (29 total documented attempts among 18 participants)	
Hanging	7
Poison	12
Other	1
Decline to answer	13

## Data Availability

The data underlying this study contain potentially identifying or sensitive information and cannot be made publicly available due to ethical restrictions. Participants did not consent to the public sharing of their data. De-identified excerpts supporting key findings may be made available upon reasonable request to the corresponding author.
